# Early Diffusion of SARS-CoV-2 Infection in the Inner Area of the Italian Sardinia Island

**DOI:** 10.3389/fmicb.2020.628194

**Published:** 2021-02-12

**Authors:** Giovanna Piras, Nicole Grandi, Maria Monne, Rosanna Asproni, Tatiana Fancello, Maura Fiamma, Giuseppe Mameli, Gavino Casu, Iana lo Maglio, Angelo D. Palmas, Enzo Tramontano

**Affiliations:** ^1^UOC Ematologia, P.O. “San Francesco,” Azienda Tutela Salute, ASSL Nuoro, Nuoro, Italy; ^2^Laboratory of Molecular Virology, Department of Life and Environmental Sciences, University of Cagliari, Cagliari, Italy; ^3^UOC Cardiologia, P.O. “San Francesco,” Azienda Tutela Salute, ASSL Nuoro, Nuoro, Italy; ^4^UOC Laboratorio Analisi Clinico-Chimiche e Microbiologia, P.O. “San Francesco,” Azienda Tutela Salute, ASSL Nuoro, Nuoro, Italy; ^5^Istituto di Ricerca Genetica e Biomedica, Consiglio Nazionale delle Ricerche, Cagliari, Italy

**Keywords:** SARS-CoV-2, Sardinia Island, pandemic, epidemiology, phylogeny, genome sequencing, COVID-19, molecular characterization

## Abstract

**Background:**

Severe acute respiratory syndrome coronavirus 2 (SARS-CoV-2) has been responsible for the coronavirus disease 2019 (COVID-19) pandemic, which started as a severe pneumonia outbreak in Wuhan, China, in December 2019. Italy has been the first European country affected by the pandemic, registering a total of 300,363 cases and 35,741 deaths until September 24, 2020. The geographical distribution of SARS-CoV-2 in Italy during early 2020 has not been homogeneous, including regions severely affected as well as administrative areas being only slightly interested by the infection. Among the latter, Sardinia represents one of the lowest incidence areas likely due to its insular nature.

**Methods:**

Next-generation sequencing of a small number of complete viral genomes from clinical samples and their virologic and phylogenetic characterization was performed.

**Results:**

We provide a first overview of the SARS-CoV-2 genomic diversity in Sardinia in the early phase of the March–May 2020 pandemic based on viral genomes isolated in the most inner regional hospital of the island. Our analysis revealed a remarkable genetic diversity in local SARS-CoV-2 viral genomes, showing the presence of at least four different clusters that can be distinguished by specific amino acid substitutions. Based on epidemiological information, these sequences can be linked to at least eight different clusters of infection, four of which likely originates from imported cases. In addition, the presence of amino acid substitutions that were not previously reported in Italian patients has been observed, asking for further investigations in a wider population to assess their prevalence and dynamics of emergence during the pandemic.

**Conclusion:**

The present study provides a snapshot of the initial phases of the SARS-CoV-2 infection in inner area of the Sardinia Island, showing an unexpected genomic diversity.

## Introduction

Severe acute respiratory syndrome coronavirus 2 (SARS-CoV-2) is a non-segmented, enveloped virus with a single-stranded, positive-sense RNA genome that has been responsible for the coronavirus disease 2019 (COVID-19) pandemic. The latter began as a severe pneumonia outbreak in Wuhan city (Hubei Province, China) in December 2019. A viral etiology of the disease was suggested, and subsequent analyses confirmed the presence of an infectious agent classified as a member of the β-coronaviruses, being highly related to the Sarbecovirus SARS-CoV that has been responsible for an epidemic outbreak in China between 2002 and 2004 (Zhu et al., 2020). Accordingly, clinical features of the disease include fever, cough, and myalgia, with the development of bilateral pneumonia and acute respiratory syndrome in severe manifestations. Epidemiological investigation highlighted that a remarkable proportion of suspected cases was linked to the exposure to live wild animals in a local seafood market, even if subsequent analyses suggested an earlier arrival of the virus (Jin et al., 2020). In the following months, SARS-CoV-2 has infected more than 40.4 million individuals worldwide, including 1,119,283 deaths as of October 20, 2020, based on the European Centre for Disease Prevention and Control^1^.

**FIGURE 1:**
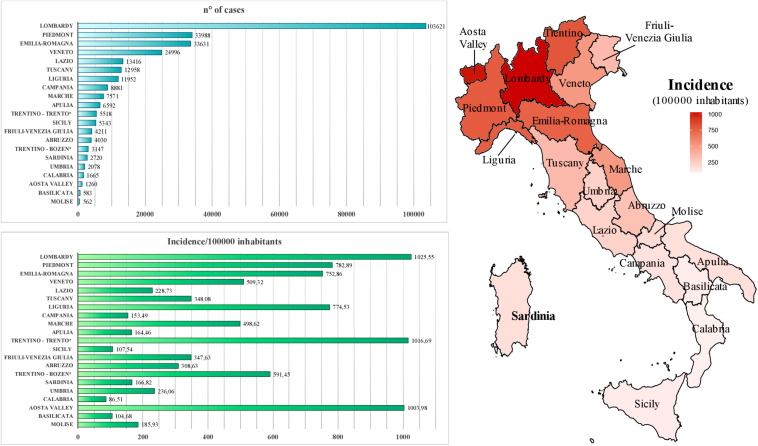
Geographic diffusion of coronavirus disease 2019 (COVID-19) infection in Italy. Autonomous provinces are marked with a *. NW, North-West; NE, North-East; C, Center; S, South; IS, Island. Epidemiological data source: ISS, https://www.iss.it/coronavirus, updated on September 15, 2020.

**FIGURE 2:**
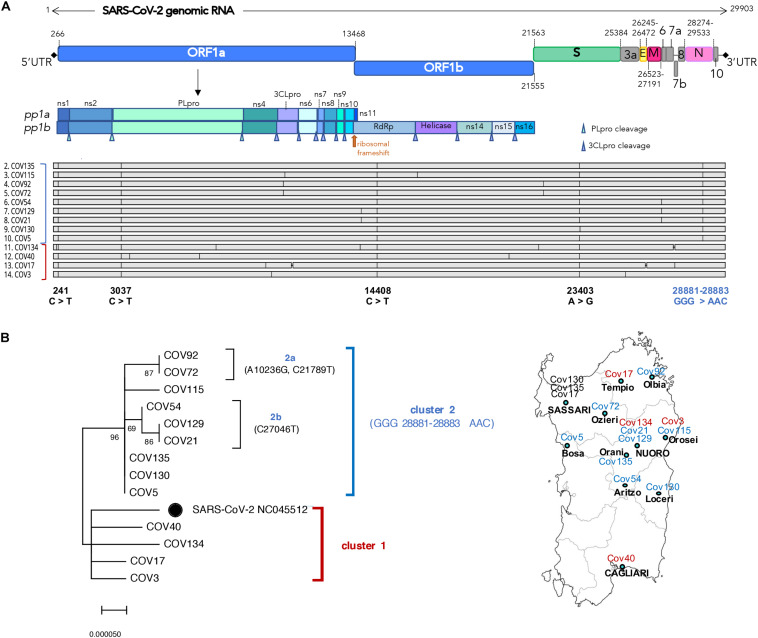
Structural and phylogenetic analysis of the 13 Nuoro severe acute respiratory syndrome coronavirus 2 (SARS-CoV-2) genomes. **(A)** Alignment of the 13 viral genomic sequences with respect to SARS-CoV-2 reference genome NC_045512.2 annotated for the position of viral genes (green labels) and their coding DNA sequence (CDS, yellow labels). For the genomic portion encoding ORF1ab polyprotein, the final individual proteins are annotated with white labels. **(B)** Neighbor-joining tree of the 13 viral genomic sequences and SARS-CoV-2 reference genome (black dot): the percentages of trees in which the associated taxa clustered together (bootstrap test, 100 replicates) are shown above the nodes. The two main clusters—named here 1 and 2—are highlighted with red and blue brackets, respectively, and the correspondent color is also indicated near the aligned sequences. The four nucleotide substitutions shared by all the sequences and the one specifically characterizing cluster 2 members are reported below the alignment in black and blue, respectively. A map of Sardinia showing the geographic distribution of samples is also provided.

Within Europe, Italy has been the earliest country affected by the pandemic. Particularly, the first cases of COVID-19 on the national territory were registered in Lombardy region, being a tourist couple from Wuhan (as first imported cases) and then a 38-year-old man from Codogno city, who represented the primary autochthonous diagnosis on February 20, 2020 (Prezioso et al., 2020). In the following months, a total of 300,363 cases have been reported, causing overall 35,741 deaths with a lethality rate of 11.9% (accessed on September 24, 2020^2^). The amount of infected individuals is probably underestimated, considering that—as in other countries—the reported number of cases in the various Italian regions is likely biased by the different amounts of diagnostic tests as well as the presence of asymptomatic or mild cases (Deng et al., 2020), likely leading to an overestimated lethality rate. During the course of the pandemic, the daily growth in the number of cases has overburdened clinical care facilities, leading to the progressive introduction of “social distancing” measures culminated with the establishment of a national lockdown (March 9–May 3, 2020) to contain virus transmission. Concerning COVID-19 distribution, the diffusion of the infection has not been homogeneous before and after the lockdown but showed a geographical gradient, with northern regions being severely affected since the very early phases (e.g., Lombardy, Veneto, and Piedmont) and administrative areas in the Center-South part of the country being only slightly interested by the infection (Figure 1) (Prezioso et al., 2020). Among the latter, Sardinia and Sicilia major islands represent two of the lowest incidence areas, with overall 166.8 and 107.5 cases each 100,000 inhabitants, respectively, until September 15, 2020 (Figure 1). It is likely that the geographical conformation of these areas contributed to the minor diffusion of the infection due to the more complex nature of the movements to/from islands, even if—for a short early period—such circulation into Sardinia island was increased, especially due to Sardinian citizens returning home as well as northern Italian inhabitants moving out of their regions in safer areas before the circulation between Italian administrative areas had been blocked by specific government measures. Accordingly, during the period from the above first SARS-CoV-2 Italian case (February 21, 2020) to the end of the national lockdown (May 3, 2020), Sardinia registered a total of 1,319 cases against the 77,528 of Lombardy (the most affected Italian area), being the fifth lower affected region in Italy (data from Italian Civil Protection’s daily bulletin, May 3, 2020). Such a limited diffusion of the infection within Sardinia territory likely led to a different epidemiological scenario with respect to non-insular areas of Italy, expecting less diversity in local viral genomes as compared to the ones circulating in highly exposed parts of the country. To assess this hypothesis, the present study took into account a total of 13 complete SARS-CoV-2 genomes, as sequenced from nasopharyngeal swabs during clinical screening at “San Francesco” Hospital laboratory for COVID-19 in Sardinia (March 28–June 15, 2020) located in Nuoro, an inner town of the Sardinia Island (Figure 2). These sequences have been analyzed in terms of viral composition and phylogeny with respect to national and international SARS-CoV-2 genomes in order to characterize them and try to hypothesize their route of arrival in the island. Contrarily to expectations, results revealed a remarkable variability even in such a small sample, providing a picture of SARS-CoV-2 genome diversity in inner Sardinia and pointing out the prevalence of imported return-associated infections in the early phase of diffusion in the island.

## Materials and Methods

### Samples

Between March 28 and June 15, 2020, nasopharyngeal swabs collected from suspected cases with or without symptoms of COVID-19 were submitted for molecular diagnosis of SARS-CoV-2 to “San Francesco” Hospital laboratory for COVID-19 in the town of Nuoro, Sardinia (Italy). On June 15, 2020, 59 samples resulted positive among 6,500 molecular tests. The 13 SARS-CoV-2 complete genomic sequences taken into account in the present study were obtained in the period between March 28 and April 28, 2020, from selected patients among the above dataset, whose characteristics are reported in Table 1. Of note, these 13 samples represent the 50% of positive cases confirmed in the considered time period (26 in total) and were selceted for sequencing based on their higher CT value. All samples were collected as part of clinical diagnostics following official procedure (ISS Working Group Diagnostics and Microbiological Surveillance of COVID-19^3^). The obtained SARS-CoV-2 genomic sequences were registered in GISAID with the following accession numbers: EPI_ISL_613560, EPI_ISL_613706, EPI_ISL_613710, EPI_ISL_ 613953, EPI_ISL_637109, EPI_ISL_613955, EPI_ISL_614396, EPI_ISL_614397, EPI_ISL_614398, EPI_ISL_458084, EPI_ISL_ 614889, EPI_ISL_637107, EPI_ISL_637108.

**TABLE 1:** Characteristics of the 13 Nuoro SARS-Cov-2 patients.

ID	Cov3	Cov5	Cov17	Cov21	Cov40	Cov54	Cov72	Cov92	Cov115	Cov129	Cov130	Cov134	Cov135
**Sex/Age**	F29	F67	F47	F62	M89	F56	F70	M95	M77	F62	F47	F75	F54
**Area**	Orosei	Bosa	Tempio	Nuoro	Cagliari	Aritzo	Ozieri	Olbia	Orosei	Nuoro	Loceri	Nuoro	Orani
**^*a*^D.o.C. (2020)**	4/04	15/04	8/04	10/04	23/04	9/04	23/04	28/04	3/04	3/04	28/04	28/04	29/03
**^*b*^Access**	External	Patient	External	External	Patient	External	Patient	Patient	External	Patient	External	External	Patient
**Contact of exposure to SARS-Cov-2**	Possible infection in United Kingdom	Contact with Sassari hospital worker infected at a congress in Northern Italy (Milan)	Close contact to a congress attendant infected in Northern Italy-(Verona)	Cohabitant of Cov129 (relatives)	Unspecified community transmission	Contact with 1st case in Ogliastra area (East Sardinia, dead for COVID-19)	Ozieri hospital worker infected at a congress in North Italy	Unspecified community transmission	Unspecified community transmission	Unspecified community transmission	Sassari hospital worker (contact with another hospital worker infected at a congress in Northern Italy, Milan)	Contact with 1st case in Nuoro (close relative, dead for COVID19)	Sassari hospital worker (contact with another hospital worker infected at a congress in Northern Italy, Milan)
**^*c*^CT (S gene)**	27	26,8	31	22	18	26	17,8	25,8	25,8	28,6	13	27	23
**^*d*^ Coverage (%) and depth (#reads)**	99.6 6,809	99.5 5,862	98.9 688	99.19 7,660	98.9 8,800	96.71 6,349	98.32 16,401	99.45 4,338	99.43 10,519	98.45 4,365	98.22 11,778	89.35 841	98.58 4,005
													

*Information includes: ^*a*^date of collection (D.o.C.); ^b^type of access to sampling, where “patient” indicated hospitalized patients, while “external” indicated external accesses; ^*c*^CT value of S gene from 2019-nCoV nasopharynx swab; ^*d*^Coverage (% of reads) and average depth (number of reads) of SARS-CoV-2 genome sequencing.*

### Extraction of RNA and Reverse Transcription Real-Time PCR

Nasopharyngeal swabs were collected and placed in 3 ml of Universal Transport Medium (UTM, Copan Universal Transport Medium), transported to the laboratory at room temperature and tested for SARS-CoV-2 the same day. Viral RNA was automatically extracted from 250 μl swabs medium using Seegene Nimbus system with the STARMag Universal Cartridge kit and tested subsequently using the Korea Ministry of Food and Drug Safety-approved Allplex 2019-nCoV assay (Arrow Diagnostics S.r.l., Genova, Italy), which detects the three target genes in a single-tube assay (E gene, RdRP gene, and N gene) as in the WHO-recommended protocols. Viral RNA aliquots from positive nasopharyngeal swabs were validated with the *RealStar SARS*-*CoV*-*2* RT-PCR Kit 1.0, which detects S gene. Ten nanograms of RNA (>1,000 virus genome copies) were used for whole viral genome sequencing.

### Next-Generation Sequencing

Libraries were prepared with the Ion AmpliSeq Library Kit Plus according to the manufacturer’s instruction using Ion AmpliSeq SARS-CoV-2 RNA custom primers panel (ID: 05280253, ThermoFisher Scientific). The RNA library preparation included reverse transcription using SuperScript VILO cDNA Synthesis Kit (ThermoFisher Scientific) with subsequently 16–21 cycles of PCR amplification on Ion Chef. Next-generation sequencing (NGS) reactions were run on Ion Torrent GeneStudio S5 sequencer.

Sequence alignments to the SARS-CoV-2 isolate Wuhan-Hu-1, complete genome (NCBI nucleotide collection, accession number: NC_045512) (Wu et al., 2020), was performed within the Torrent Server of Ion Torrent S5 sequencer using default settings. The aligned reads were utilized for both reference-guided assemblies. Assembly was performed using the Iterative Refinement Meta-Assembler (IRMA) v.0.6.1 (Shepard et al., 2016) that produced a consensus sequence for each sample using a >50% cutoff for calling single-nucleotide polymorphisms. IRMA utilizes multiple steps of alignment, variant calling, and consensus building by capitalizing on multiple allele frequency confidence intervals and read depth. Aligned reads were validated through the Integrative Genomics Viewer (IGV) v.2.5.3 (Thorvaldsdóttir et al., 2013).

### Structural Characterization of SARS-CoV-2 Genomes

The 13 SARS-CoV-2 complete genomes have been aligned with respect to SARS-CoV-2 reference isolate Wuhan-Hu-1 (NCBI nucleotide collection, accession number: NC_045512) annotated with the positions of viral genes and corresponding proteins. The alignment has been used for both structural and phylogenetic characterization with respect to the reference genome. The 13 SARS-CoV-2 complete genomes coding sequences have then been bioinformatically translated to analyze the resulting proteins as compared to the reference ones, with particular attention to amino acid substitutions known to impact protein activity and structure. All pairwise and multiple alignments have been performed using MAFFT algorithm (Katoh and Standley, 2013) and have been visually inspected and, if necessary, manually improved on Geneious Prime platform (Kearse et al., 2012). The global distribution and prevalence of each amino acid substitution have been evaluated using the Nextstrain genomic epidemiology site^4^ and the Tracking Mutations tool as part of the COVID-19 viral genome analysis pipeline from Los Alamos National Laboratory^5^.

### Retrieval of Publicly Available SARS-CoV-2 Genomic Sequences

In order to further characterize the phylogeny of the 13 SARS-CoV-2 genomic sequences generated in the study, we analyzed them with respect to a dataset of SARS-CoV-2 complete genomes from all over the world, available in GISAID repository^6^ (accessed at the beginning of July 2020). Particularly, we included in our analyses all GISAID sequences representing complete viral genomes (excluding hence partial sequences coding for individual proteins or polyproteins), removing the ones obtained from non-human hosts or containing stretches of N (likely due to low coverage portions), thus ending up with a final dataset of about 59,700 SARS-CoV-2 genomic sequences, among which 160 were obtained from Italian patients.

### Phylogenetic Analyses

**FIGURE 3:**
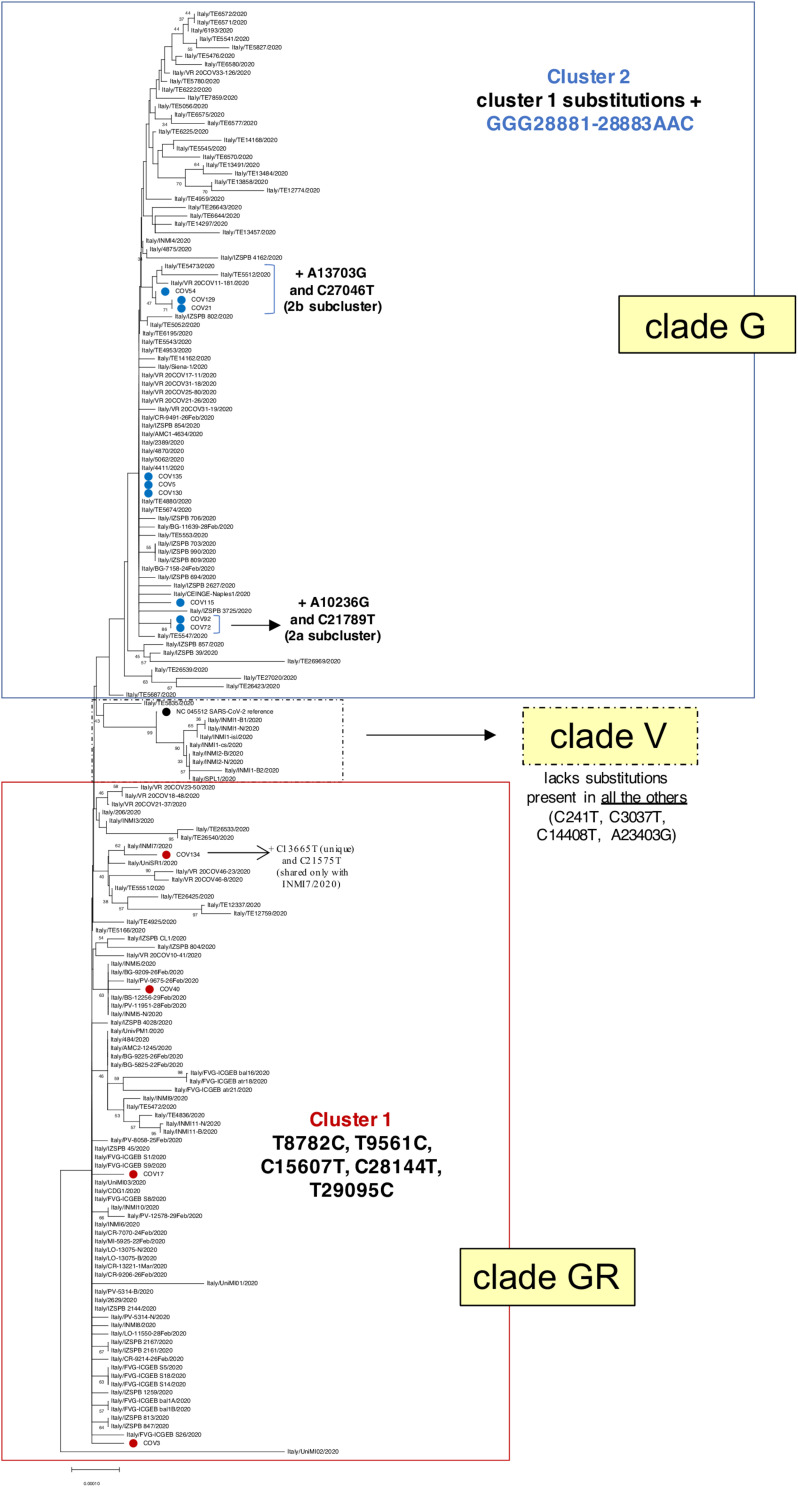
Phylogenetic analysis of Nuoro severe acute respiratory syndrome coronavirus 2 (SARS-CoV-2) sequences and GISAID viral genomes from Italy. Viral genomic sequences identified in Italy have been retrieved from GISAID and analyzed in a neighbor-joining tree. The percentages of trees in which the associated taxa clustered together (bootstrap test, 100 replicates) are shown next to the branches. Samples generated in this work are marked with red and blue dots, depending on their belonging to cluster 1 (clade GR) or cluster 2 (clade G), respectively. SARS-Cov-2 reference genome NC_045512.2 is indicated with a black dot. For the sake of clarity, bootstrap values lower than 30% have been hidden.

Nucleotide identity of the 13 SARS-CoV-2 genomic sequences generated in this study with respect to NC_045512 reference sequence has been calculated with Mega X Software (Kumar et al., 2008) using p-distance model and applying pairwise deletion option. Phylogenetic trees for the 13 SARS-CoV-2 genomic sequences generated in this study with NC_045512 reference sequence (Figure 2) and with the ensemble of GISAID SARS-CoV-2 sequences from Italy (Figure 3) have been built from multiple nucleotide alignments (see above) using Mega X Software (Kumar et al., 2008) and applying both neighbor-joining and maximum likelihood statistical methods with the pairwise deletion option. Phylogenies were tested by the bootstrap method with 1,000 and 100 replicates, respectively.

Maximum likelihood phylogenetic tree for the 13 SARS-CoV-2 genomic sequences generated in this study with the ensemble of GISAID SARS-CoV-2 sequences from all the world and the subset of European ones (Supplementary Files 1, 2, respectively) has been inferred with FastTree software, which allows to compute approximately maximum likelihood trees for very large alignments in a reasonable time, applying a generalized time-reversible (GTR) model of nucleotide evolution and CAT approximation for the varying rates of evolution across sites. Local support values have been computed with the Shimodaira–Hasegawa test. Trees have been visualized and analyzed with Dendroscope, v.3.7.2 (Huson et al., 2007).

## Results

### Next-Generation Sequencing of SARS-CoV-2 Genomes in Clinical Samples

The present study took into account a total of 13 SARS-CoV-2-positive patients who were tested through a nasopharyngeal swab at “San Francesco” Hospital (Nuoro, Sardinia, Italy) in the early phase of the SARS-CoV-2 pandemic (March 28–April 28, 2020), and that will be termed here as “Nuoro sequences.” Of note, these 13 samples represent the 50% of positive cases confirmed in Nuoro inner area at the considered time period (26 in total) and were selected for sequencing based on the fact that they showed the highest CT value as measured by Real Time PCR on the S gene. The patients’ characteristics are summarized in Table 1, along with the correspondent CT value as well as the sequencing depth and coverage (in million and percentage of reads, respectively). In line with the virus diffusion among elderly persons, patients median age was 62 (range 29–95), and the majority of patients were women (10/13). Concerning the geographical area of origin, most of the patients came from the North- and Central-East part of the island, with the exception of Cov40 who came from Cagliari (South Sardinia). As reported in the exposure data, four out of 13 individuals (Cov5, Cov72, Cov130, and Cov135) referred to have had contacts with positive health care workers, who were infected while attending scientific congresses in Milan, Lombardy (Northern Italy), individuating hospitals as the main place of transmission. Similarly, Cov17 was infected by a relative who attended a scientific congress that took place in Verona, Veneto (Northern-East Italy), while Cov3 reported to have likely acquired the infection during a 2-month stay in London, United Kingdom. Another proportion of patients referred to have not moved from Sardinia in the weeks before sampling, representing hence local community transmissions. Among the latter, two patients (Cov21 and Cov129) are cohabiting relatives and had likely a common exposure to the infection. Cov54 comes from Ogliastra area, i.e., the central-East part of the island, and referred to have had contacts with the first (deadly) case of COVID-19 in this area. Similarly, Cov134 is known to have close contact with a relative accounted as the first (deadly) case of COVID-19 in the town of Nuoro. No additional information was indeed available for the remaining patients (Cov40, Cov92, Cov115), representing thus not better specified community transmissions.

### Phylogenetic and Structural Analysis of Nuoro SARS-CoV-2 Sequences

Firstly, the 13 SARS-CoV-2 genomic sequences have been compared in terms of nucleotide sequence and phylogeny with respect to SARS-CoV-2 isolate Wuhan-Hu-1 (NC_045512) (Wu et al., 2020) that has been used as reference viral genome for all subsequent analyses (Figure 2). As shown in the alignment (Figure 2A), all the sequences harbor some mutations with respect to the reference, having overall 99.96% identity to it (identity values ranging from 99.93 to 99.98%, data not shown). Interestingly, all the 13 Nuoro SARS-CoV-2 sequences show some common single-nucleotide substitutions in the 5′UTR (241 C > T) and in the genic regions coding for the papain-like protease (PL-pro) (*nsp3*, 3,037 C > T), the RNA-dependent DNA polymerase (RdRp) (*nsp12*, 14,408 C > T), and the spike protein (*S*, 23,403 A > G) (Figure 2A). A subset of sequences also sharesan additional mutation involving three consecutive nucleotides in the nucleocapsid phosphoprotein gene (*N*, 28,881–28,883 GGG > AAC).

In order to gain more insights into the phylogenetic relationships among Nuoro SARS-CoV-2 sequences, we generated a neighbor-joining tree, observing the presence of two distinct main clusters named here 1 and 2 (Figure 2B). Of note, all members of cluster 2 share the above 28,881–28,883 GGG > AAC mutations in N gene (96% bootstrap support), while cluster 1 sequences are more related to SARS-CoV-2 reference. Furthermore, cluster 2 includes two subclusters, named here 2a (Cov72 and Cov92, 87% bootstrap) and 2b (Cov21, Cov54, and Cov129, 69% bootstrap) that groups viral genomes sharing additional nucleotide substitutions not present in any other sequence (Figure 2B). As expected from the fact that Cov21 and Cov129 had a common exposure to the infection, their viral genomic sequences also show a supported phylogenetic relation and were highly similar to Cov54 as well. Also, Cov5, Cov130, and Cov135 had the same nucleotide sequence, possibly representing a unique cluster of infection. Of note, comparable results have also been obtained using the maximum likelihood method (Supplementary Figure 1).

### Characterization of the 13 Nuoro SARS-CoV-2 Genome Substitutions as Compared to SARS-CoV-2 Reference

The nucleotide variations observed in the alignment of the 13 SARS-CoV-2 genomes as compared to Wuhan reference isolates have been further characterized for their effect on the amino acid sequence and predicted impact on the protein activity (Table 2). Overall, 26 different nucleotide mutations have been observed, of which 14 (54%) led to amino acid changes in the correspondent protein. As already mentioned, four nucleotide substitutions are common to all the 13 SARS-CoV-2 sequences and have been reported to co-evolve with high prevalence in European SARS-CoV-2 genomes (Liu et al., 2020; Lorusso et al., 2020), being characteristics of SARS-CoV-2 clade G (Table 2). Of these, one is in the 5′UTR and another is a synonymous substitution for phenylalanine (*nsp3* gene, F924F), while the other two led to amino acid substitutions in RdRp (P4715L) and S protein (D614G). Interestingly, both these amino acid substitutions have become dominant in global (86.77 and 85.57%, respectively) and Italian (93.63 and 97.51%, respectively) SARS-CoV-2 genomes (Table 2) and have also been reported to impact viral pathogenesis. In fact, the change of Proline to Leucine in RdRp is associated with epitope loss that may cause antibody escape variants, and it has been proposed to increase the mutation rate and the transmissibility of the virus (Gupta and Mandal, 2020). The replacement of S protein aspartate with glycine is indeed known to be associated with increased fatality rate and suggested to enhance furin cleavage efficiency and viral infectivity (Becerra-Flores and Cardozo, 2020; Gobeil et al., 2020; Shi et al., 2020). In addition to the above clade G mutations, a triple contiguous nucleotide mutation in the *N* gene is characteristic of cluster 2 members and has been firstly reported in an isolate from a North European patient with a travel history to Italy (Lorusso et al., 2020) (Table 2). It led to the substitution of two consecutive amino acids (RG203-204KR), with a significant change in the isoelectric point (Pachetti et al., 2020), and it is characteristic of SARS-CoV-2 clade GR. The alternative amino acids at these positions are now quite common in the Italian population, being found in about half of the total viral sequences (Table 2).

**TABLE 2:** Analysis of the 13 Nuoro SARS-CoV-2 genome substitutions as compared to SARS-CoV-2 reference.

SARS2 position	187	241*	3037*	3415	5866	6526	7267	9479	9724	10236	10323	13665	13703	14408*	16203	20268	21557	21789	23403*	25429	27046	27568	27622	28881–28883
**SARS2 Base**	**A**	**C**	**C**	**A**	**C**	**A**	**C**	**G**	**C**	**A**	**A**	**C**	**A**	**C**	**T**	**A**	**C**	**C**	**A**	**G**	**C**	**TTTA GCAC TCAA**	**T**	**GGG**
COV003		T	T						T					**T**					**G**	**T**				
COV005		T	T											**T**					**G**					**AAC**
COV017		T	T					**T**						**T**					**G**				**G**	
COV021		T	T										**G**	**T**					**G**		**T**			**AAC**
COV040		T	T	G		T								**T**		G			**G**					
COV054		T	T											**T**					**G**		**T**			**AAC**
COV072		T	T							**G**				**T**			C/T	**T**	**G**					**AAC**
COV092		T	T							**G**				**T**				**T**	**G**					**AAC**
COV115		T	T								**G**			**T**	C				**G**					**AAC**
COV129		T	T		C/T								**G**	**T**					**G**		**T**			**AAC**
COV130		T	T											**T**					**G**					**AAC**
COV134	G	T	T				T					T		**T**					**G**			**del**		
COV135		T	T											**T**					**G**					**AAC**

**Genome region**	**5’UTR**		***ORF1ab***														***S* gene**			***ORF 3a***	**M gene**	**ORF7a**		**N gene**
	**Leader**	**SL5b loop**	**Nsp3**					**Nsp4**		**Nsp5 (3CL-pro)**		**Nsp12 (RdRp)**				**Nsp15**	**Spike S1 subunit**			**Viro- porin 3a**	**Membrane glyco protein**	**TM protein**		**Nucleo capsid**
												
**Effect on protein sequence**	up stream	up stream	F 924 F	K 1050 K	F 1867 F	T 2087 T	F 2334 F	**G 3072 C**	F 3153 F	**K 3324 R**	**K 3353 R**	H 4467 H	**N 4480 S**	**P 4715 L^1^**	Y 53 13 Y	L 66 68 L	up stream	**T 76 I**	**D 614 G^2^**	**V 13 L**	**T 175 M**	**S 60 fs**	**L 77 V**	**R 203 K G 204 R**

**cluster**		1 and 2	1 and 2							2a				1 and 2				2a	1 and 2		2b			2
**Global prevalence (%)**								0.23		0.04	0.66		0	86.77				0.06	85.57	1.60	0.82	0.02	NR	41.65 41.29
**Italian prevalence (%)**								NR		NR	NR		0.75	93.63				NR	97.51	NR	1.12	NR	NR	47.57 47.57

*Nucleotide substitutions and other mutations identified as compared to SARS-CoV-2 reference genome sequence NC_045512.2 are reported along with their effect on the correspondent protein product. Substitutions at nucleotide positions indicated with a “^∗^” are known to co-evolve and are prevalent in European viral genomes. Mutations leading to amino acid changes in the correspondent protein are indicated in bold, and their global prevalence has been calculated based on the sequences reported by GISAID (2019-12-24–2020-10-07) and took into account by the Tracking Mutation tools of Los Alamos National Laboratory COVID-19 viral genome analysis pipeline (https://cov.lanl.gov). NR, not reported. ^1^Proposed to increase mutation rate and transmissibility. ^2^Mutation is known to be associated with increased fatality rate.*

As previously mentioned, part of cluster 2 members can be further divided into two subclusters carrying additional substitutions. Particularly, the two additional mutations characteristic to 2a subcluster have been found rarely at the global level (0.04–0.06% of prevalence) and are not reported at all among Italian patients (Table 2). In fact, K3324R substitution in 3C-like protease (3CL-pro or nsp5) has been reported in a minority of patients in the South area of Asia (Malaysia and Hong Kong). Similarly, besides the common mutation D614G, subcluster 2a Spike protein presents the additional substitution T76I, which is found in a minority of viral genomes from Turkey, Saudi Arabia, and Brazil (global prevalence = 0.06%) (Table 2). Concerning subcluster 2b, the missense mutation T175M in the membrane glycoprotein (M) has already been reported to arise following the acquisition of RG203-204KR substitution (Liu et al., 2020), being found in a minority of samples from different European countries. Interestingly, the prevalence of this substitution in Italian viral genomes is higher than the global one (1.12% vs. 0.82%). Furthermore, a proportion of the observed non-synonymous substitutions are not associated with a particular phylogenetic cluster, being found in one or few patients and mostly not reported in GISAID Italian SARS-CoV-2 genomes (Table 2). For example, Nsp4 G3072C (Cov17) has been previously reported in Tunisia and in some parts of East Europe and South America (global prevalence = 0.23%); Nsp5/3CL-pro K3353R (Cov115) is found mostly in Iceland and Ireland (global prevalence = 0.66%), while V13L substitution in ORF3a viroporin (Cov3) is relatively diffused in some patients from North and East Europe (global prevalence = 1.60%) (Table 2). Similarly, the single nucleotide deletion leading to a frameshift in *ORF7a* gene (Cov134) is limited to German patients among European cases (global prevalence = 0.02%), while an exception is represented by N4480S (nsp12/RdRp, Cov21, and Cov129), being instead reported in Italy only with a prevalence of 0.75%. Also Spike L to F mutation at residue 5 (Cov134) is reported both in Italian and global sequences, with prevalence rates of 0.61 and 1.01%, respectively. Indeed, ORF7a L77V substitution (Cov17) has not been previously reported in any of the available viral genomes (Table 2). Finally, in two cases, sequencing profiles highlighted the presence of an emerging single nucleotide mutation, with the substitution of a C with a T in Cov129 *ORF1ab nsp3* gene and upstream Cov72 *S* gene (position 5866 and 21557 of the reference SARS-CoV-2 genome sequence). While the former represents a synonymous substitution (F1867F), the latter introduces a stop codon, which is, however, located outside the coding portions of the flanking genes.

### Phylogenetic Analysis of Nuoro SARS-CoV-2 Sequences With Respect to the SARS-CoV-2 Pandemic

In order to contextualize the analyzed sequences with respect to the viral strains circulating during the SARS-CoV-2 pandemic, we analyzed the 13 Nuoro SARS-Cov-2 genomes with respect to a set of about 59,700 complete viral genomes from all over the world, as downloaded from GISAID repository (see footnote 6, accessed at the beginning of July 2020).

As a first step, we inferred a neighbor-joining phylogenetic tree including 160 genomes from Italian SARS-CoV-2 patients, as extracted from the above GISAID dataset (Figure 3). Of note, the presence of the two identified main clusters was confirmed also in this tree, corresponding to clades GR (cluster 1) and G (cluster 2), as already suggested by the amino acid substitutions observed in the different genomic sequences (Table 2). In fact, Nuoro cluster 2 sequences are grouped in the upper part of the tree, with other Italian SARS-CoV-2 variants belonging to clade G and sharing the additional triple substitution at nucleotides 28881–28883 (Figure 3). Also, in this cluster, 2a and 2b subclusters can be clearly distinguished. Particularly, 2a members (Cov72 and Cov92) are included in a phylogenetic cluster (87% bootstrap support) in line with the presence of 2a characteristics substitutions, which are absent in all other sequences. Subcluster 2b indeed includes Cov21, Cov129, and Cov54 (68% bootstrap support)—as already seen in the first phylogenetic tree—as well as some viral genomes from Verona (Veneto) and Teramo (Abruzzo) (Figure 3). These sequences share with Nuoro SARS-CoV-2 subcluster members the characteristic mutation C27046T, which is not found in the other Italian viral genomes. In addition to clusters 1 and 2, a third phylogenetic cluster is present in the three, embedded within clade GR, and corresponds to clade V (Figure 3). The latter includes viral genomes sharing a higher identity to SARS-CoV-2 reference due to the lack of some nucleotide substitutions found in all the other Italian sequences and group in fact with the reference supported by a 94% bootstrap value (Figure 3). Comparable results have been obtained using the maximum likelihood method (Supplementary Figure 1).

**FIGURE 4:**
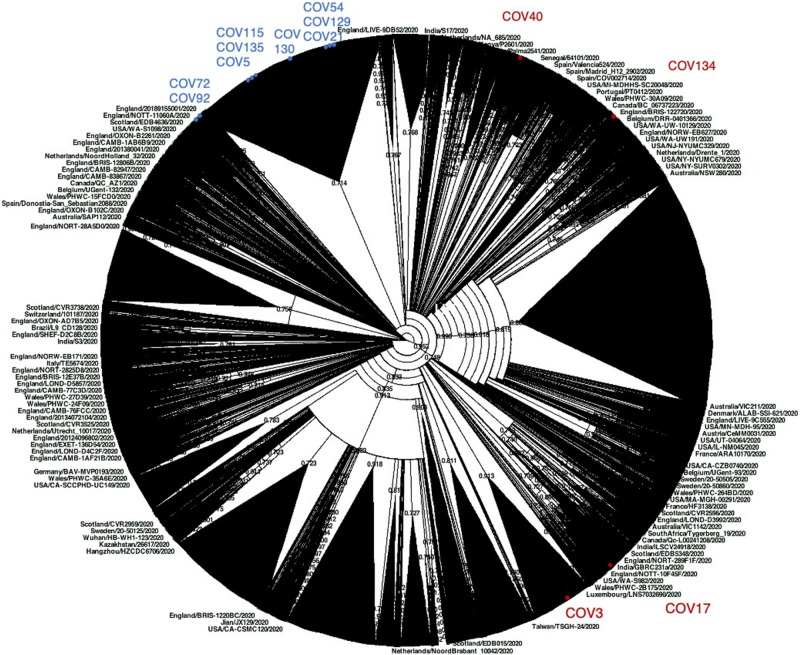
Phylogenetic analysis of Nuoro severe acute respiratory syndrome coronavirus 2 (SARS-CoV-2) sequences and GISAID worldwide viral genomes. About 59,700 viral genomic sequences identified globally have been retrieved from GISAID and analyzed in a maximum likelihood tree. Samples generated in this work are marked with red and blue dots, depending on their belonging to cluster 1 (clade GR) or cluster 2 (clade G), respectively. The corresponding source tree in Newick format is also provided as Supplementary File 1 to allow its detailed inspection in freely available tree viewers.

Then, willing to assess the phylogenetic behavior of the 13 Nuoro SARS-Cov-2 genomes with respect to the global SARS-CoV-2 variability, we generated a maximum likelihood tree including around 60,000 viral genomes available in GISAID repository (Figure 4). Due to the dimension of the tree, which unavoidably compromises the figure clarity, we also provided the source tree in Newick format (Supplementary File 1) in order to allow its detailed inspection in freely available tree viewers such as Dendroscope (Huson et al., 2007). The tree confirmed the division of Nuoro SARS-CoV-2 sequences into two separate phylogenetic clusters, each grouping with clade GR (cluster 1) or clade G (cluster 2) viral genomes from all over the world. Particularly, the distribution of Nuoro SARS-CoV-2 genomes within the tree resembles the clusters observed in previous trees (Figure 4), with a clear division between cluster 1 and cluster 2 members. Also, in this tree, cluster 2 members appear to be included in a more condensed group, even if a clear division is present between subcluster 1 members (Cov72 and Cov91) and the rest of the sequences. This confirms the value of the identified characteristic mutations, even if they showed a poor phylogenetic support in Figure 3 tree. Contrarily, cluster 2 sequences are widespread in the tree, suggesting a higher heterogenicity within clade GR (Figure 4). Finally, all Nuoro SARS-CoV-2 sequences are found within European viral genomes, mostly from England and Northern Europe, in line with the prevalent diffusion of these clades in European countries. To further explore such relationship with European SARS-CoV-2 genomes, we generated another phylogenetic tree extracting only the European GISAID entries (about 38,130 viral sequences, included as Newick tree in Supplementary File 2) and we focused on the clusters including each Nuoro sequence. In line with what was observed in the global SARS-CoV-2 tree, Nuoro sequences grouped mainly with viral genomes from England and other Northern European countries, with support values from 70 to 99%. In detail, in line with the context of exposure during a travel to London, Cov3 formed a cluster with sequences from a relatively enclosed area of United Kingdom (Cambridge, England, 92% support). Also, Cov115 formed a cluster with a group of localized sequences, all from the Northern part of Ireland (74% support). All the patients who had contact with attendants to scientific congresses in Northern Italy (Cov5, Cov17, Cov72, Cov130, and Cov135) clustered with sequences from United Kingdom, including England, Wales, and Northern Ireland. Particularly, Cov17 formed a small cluster with viral sequences from Nottingham, England (3) and Cardiff, Wales (5) (77% support); Cov72 grouped with sequences from England (Birmingham, Bristol, and Sheffield, 94% support). Cov5, Cov130, and Cov135 were indeed included in a common, big phylogenetic group (85% support), including several genomes from England, Scotland, Wales, and Northern Ireland, plus some Portugal clusters. In support of their belonging to subcluster 2b, also Cov21, Cov129, and Cov54 were included in a common big phylogenetic cluster (92% support), forming individual subclusters with genomes mainly from the North part of Europe (Netherlands, United Kingdom, Iceland). Finally, Cov92 and Cov134 clustered independently with genome sequences from various parts of England (95 and 98% support, respectively), while Cov40 was included in a heterogeneous cluster (United Kingdom, Iceland, Spain) and grouped with a SARS-2-CoV sequence from Spain (75% support).

## Discussion

Infectious agents’ diffusion, especially in case of epidemic events, is strictly linked to the movement of people within and between the various geographical areas (Lin et al., 2020). Hence, geographical conformation and transport connections can have a significant impact on infection dynamics. This is particularly relevant in the case of places that are in geographical isolation, including remote areas and islands, which can be affected by significant limitations in the movement of people and goods as compared to other areas, likely leading to different prevalence and diversity in the circulating virus.

In the present study, we focused on 13 SARS-CoV-2 sequences representing a selection of the first complete viral genomes that were obtained in Sardinia during the early phase of the COVID-19 pandemic (March 28–April 28, 2020). According to the limited diffusion of the infection in Sardinia, in such time period, the whole island counted a total of 661 infected individuals, accounting for 0.6% of national cases. Hence, even if limited in terms of sample size, the considered population is representative of the Sardinian situation at the time—constituting overall 50% of the total positive samples identified at San Francesco Hospital until the end of April 2020—and their characteristics (prevalence of women, median age around 62 years old) are in line with the ones observed at the national level (Prezioso and Pietropaolo, 2020; Prezioso et al., 2020). Of note, epidemiological information indicates that among the 13 patients taken into account, around half (6) likely acquired the infection out of the island or from persons who returned to Sardinia from areas with highest prevalence of SARS-CoV-2, representing directly imported cases (Table 1). In fact, five individuals declared to have had contacts with positive persons who attended scientific congresses in Northern Italy (Cov5, Cov17, Cov72, Cov130, and Cov135), while Cov3 declared to have had contact with a positive individual during a travel in London, United Kingdom.

Phylogenetic and structural characterization with respect to SARS-CoV-2 reference revealed that all Nuoro SARS-CoV-2 genomes harbor some nucleotide mutations (99.93–99.98% identity) and can be classified in at least two phylogenetic clusters, corresponding to GISAID clades G and GR (Figures 2, 3). Overall, 27 different mutations have been observed, 16 of which led to amino acid changes in the correspondent proteins. Of these, Nsp12 P4715L and Spike D614G were shared by all Nuoro viral genomes, being characteristic of both G and GR clades. In addition, GR clade members held a double substitution in the two consecutive amino acids of N protein (R 203 K and G 204 R) (Figure 1 and Table 2). Among clade GR members, two further internal subclusters could be distinguished and were named 2a and 2b. The respective viral members share unique additional amino acid substitutions that are not reported or rarely found in Italian and European SARS-CoV-2 genomes. These results are in line with the presence of at least four distinct SARS-CoV-2 circulating variants that could have been present in Sardinia since the early phases of the pandemic, despite the geographical isolation and the relatively low number of local cases reported during the considered time period. The existence of multiple routes of introduction of SARS-CoV-2 strains in the island is supported also by the fact that—besides the mutation with phylogenetic significance—the analyzed viral genomes harbored various amino acid substitutions that are not associated with a particular phylogenetic cluster and were found in one or few patients, being often poorly represented in GISAID sequences (not reported in GISAID Italian SARS-CoV-2 genomes, Table 2). It will be of interest to assess if these sequences, as well as subcluster 2a and 2b rare mutations, have evolved in terms of prevalence in the subsequent phases of the pandemic and to investigate their impact on pivotal viral activities, especially when located in functional enzymatic domains and epitopes relevant to viral entry and immune recognition. For example, K3324R (subcluster 2a; 0.04 global prevalence) and the downstream K3353R (0.66 global prevalence) in 3CL-pro are not present in Italian viral sequences and fall into regions relevant for secondary structure formation (helix and β-sheet, respectively) based on the corresponding Protein Data Bank crystal structure (PDB 6M2Q; Su et al., 2020). Similarly, N4480S mutation in RdRp enzyme (global and Italian prevalence of 0 and 0.75, respectively) and L77V mutation in ORF7a TM protein (unreported in global and Italian SARS-CoV-2 genomes) occur in regions involved in helix (PDB 6M71; Gao et al., 2020) and β-strand (PDB 6W379) formations, respectively.

The phylogenetic comparison with Italian (Figure 3) and global (Figure 4 and Supplementary File 1). GISAID SARS-CoV-2 genomes confirmed the remarkable diversity of Nuoro sequences that, even if sampled in a limited area of Sardinia, were interspersed, forming phylogenetic clusters with genomes of a different geographic origin instead of being grouped in the same part of the tree. A focus on European genome phylogeny (Supplementary File 2) corroborated such hypothesis, revealing different independent clusters including Nuoro SARS-CoV-2 genomes and viral sequences obtained mainly in United Kingdom and Northern Europe, with support values from 70 to 99%. In detail, all members of subcluster 2b (Cov21, Cov54, and Cov129) confirmed their phylogenetic relation also in this tree, being included in the same big cluster with viral genomes from Northern Europe. A relation between Cov21 and Cov129 was rather expected, being relatives and living even in the same house, and in fact they share a nucleotide substitution (A10236G) absent in all the other patients. A second one (C27046T) is shared by both with Cov54 only, allowing to speculate a first, common local cluster of infection. In this regard, even if no relations between these patients are known, the fact that Cov54 referred an exposure linked to the first case of COVID-19 in the Ogliastra area, who was a physician and surely had contacts with many individuals, leaves room to speculate a possible common origin of the infection. Of note, Cov129—i.e., the first one who acquired the infection among the three—showed the emergence of an alternative nucleotide in position 5866, suggesting an evolving situation of viral quasi-species that could possibly have emerged also in the other two patients. For what concerns the patients referring an exposure linked to the contact with person who attended scientific congresses (Cov5, Cov17, Cov72, Cov130, and Cov135), these viral genomes are supposed to have been imported from the northern part of Italy. Accordingly, their viral sequences differ from the local ones, grouping with SARS-CoV-2 sequences from relatively localized areas in United Kingdom and being likely linkable to three different events of introduction in Sardinia territory. In fact, Cov17, who was referred to be infected by a relative who attended a congress in Verona city, Veneto, belongs to the G clade and clustered with SARS-CoV-2 sequences from Nottingham and Cardiff, representing a first imported case. Among the others, all classified in GR clade, Cov5, Cov130, and Cov135 referred the contact with a positive health care worker at Sassari hospital and, in line with this common place of exposure, they share identical SARS-CoV-2 viral genomes and were included in a common big cluster with sequences from England and Wales. This is likely representative of a second imported case that generated a local cluster of infection, taking advantage of the hospital environment. Cov72 referred a similar exposure, but through contact with a health care worker at Ozieri hospital who attended a different conference, and is thus derived from a third imported case, as supported by the presence of specific nucleotide substitutions (A10236G and C21789T) not present in any other patients except for Cov92, which could be likely part of the same local infection cluster. Similar to what was observed for Cov129, also Cov72 presents, in addition, the emergence of a single nucleotide variant upstream the Spike gene, in line with an earlier exposure as compared to Cov92. A fourth importation event is represented by Cov3, who referred the contact with a positive individual during a travel in London. Accordingly, Cov3 viral genome clustered with SARS-CoV-2 genomes from a very restricted area of United Kingdom, being found near some genomes from the close town of Cambridge and presenting furthermore an amino acid substitution in Viroporin 3a (V13L) that is not reported among Italian patients, but it is known to occur in United Kingdom viral genomes with a prevalence of 3.5% (Table 2). The rest of Nuoro SARS-2-CoV patients (Cov40, Cov115, and Cov134) showed some unique nucleotide substitutions that made them different from the other Nuoro SARS-CoV-2 genomes and were interspersed within European sequences in Supplementary File 2 tree, representing likely three independent transmission events. Cov40 (the only patient from Cagliari area) clustered with a Spanish SARS-2-CoV sequence (74% support) and is included in a phylogenetic group formed by viral genomes from Spain, England, Scotland, Wales, Netherlands, and Iceland; while Cov115 formed a cluster with sequences limited to the area of Northern Ireland. Finally, Cov134 is the viral genome with the highest divergence to SARS-CoV-2 reference, grouping with sequences mostly from England and forming a small subcluster with some viral genomes from Milan, England, and Bavaria. Of note, Cov134 is a close relative of the first (deadly) case of COVID-19 in this town, who referred contact with a positive person from Milan. In line with this information, Cov134 viral sequence presents a C21757T nucleotide substitution that was found only in another Italian sequence from Milan (Figure 3).

Overall, our results—even if based on a limited number of samples—provide an initial snapshot of early pandemic SARS-CoV-2 sequences circulating in Sardinia, a territory in which the movement of people and goods is strictly influenced by the insular conformation. Despite this, a remarkable genomic diversity has been observed, supporting the presence of at least eight different infection routes among the 13 samples taken into account with a main role of imported cases in the early phases of the pandemic. In particular, the combination of epidemiological information and phylogenetic and structural analyses supports the presence of a direct importation event (Cov3), three clusters of local diffusion from as many imported cases affecting professionals returning from scientific congresses (one including Cov5, Cov130, and Cov135, a second regarding Cov17 and the third one including Cov72 and Cov92), a cluster of local diffusion (Cov21, Cov129, and Cov54), and three independent local cases showing high divergence with respect to the other sequences (Cov40, Cov115, and Cov134). However, the fact that all Nuoro SARS-CoV-2 genomes form phylogenetic clusters with foreign viral sequences (mainly from Northern Europe) together with the presence of amino acid substitutions not reported in Italy leaves open the possible presence of imported cases among supposed local community transmission as well. Such a pivotal role of imported SARS-CoV-2 infections is well represented by the infection trend in Sardinia until the beginning of the pandemic (source: ISS^7^, updated on September 15, 2020). In fact, a first increase of cases has been observed before and during the first weeks of national lockdown mainly due to Sardinian citizens and northern Italian inhabitants moving in safer areas before the circulation between administrative areas had been blocked. This first peak has then been followed by a period of relatively low diffusion, reflecting the efficacy of the measures to counteract SARS-CoV-2 transmission, showing a second peak of infection increase only starting from the end of July. The latter could be linked to the concentration of touristic arrivals around the month of August, given the consolidated touristic vocation of Sardinia that attracts many Italian and foreign visitors each summer. Indeed, if comparing the number of cases reported in the first phase of the pandemic (March to mid-June) to the ones registered during the touristic season (mid-June to August), Sardinia SARS-CoV-2-infected patients have almost tripled, showing an increase from 1,363 to 3,900. However, further studies on a wider population and considering a longer time range are needed to assess the real impact of imported cases on SARS-CoV-2 epidemiology and genetic diversity in Sardinia and to evaluate the efficacy of the suspension of non-essential travels in limiting the virus diffusion in naturally low-incidence areas such as Italian islands.

## Conclusion

The present study provides an initial snapshot of SARS-CoV-2 genomic diversity during the early phases of the pandemic in Sardinia Island, which has been interested by a relatively limited diffusion of the infection in contrast to the rest of the country. Despite this, a remarkable diversity in local SARS-Cov-2 viral genomes has been characterized, being strongly influenced by the presence of numerous imported cases. In addition, phylogenetic and structural characterization revealed the presence of amino acid substitutions that were not previously reported in Italian patients, asking for further studies in a wider population to assess their prevalence and variation during the pandemic, as well as their possible impact on virus infectivity and virulence.

## Data Availability Statement

SARS-CoV-2 genome sequences analyzed in this study are deposited in GISAID database (https://www.gisaid.org) with the following accession numbers: EPI_ISL_613560, EPI_ISL_613706, EPI_ISL_613710, EPI_ISL_613953, EPI_ISL_637109, EPI_ISL_613955, EPI_ISL_614396, EPI_ISL_614397, EPI_ISL_614398, EPI_ISL_458084, EPI_ISL_614889, EPI_ISL_637107, EPI_ISL_637108; and in GenBank repository(https://www.ncbi.nlm.nih.gov/genbank/) with the following accession numbers: MT622321 (CoV129) and MW403692-MW403703.

## Ethics Statement

All samples were collected as part of clinical diagnostics following official procedure (ISS Working group Diagnostics and microbiological surveillance of COVID-19, https://www.iss.it/rapporti-covid-19//asset_publisher/btw1J82wtYzH/content/id/5329985). All samples and data collected are directly related to italian pandemic control, and were previously anonymised as required by the Italian Data Protection Code (Legislative Decree 196/2003) and the general authorisations issued by the Data Protection Authority. Ethics Committee approval was deemed unnecessary because, under Italian law, all sensitive data were deleted and we collected only age, gender, sampling date and demographic information (Art. 6 and Art. 9 of Legislative Decree 211/2003).

## Author Contributions

GP, MM, MF, and GM collected the clinical samples and epidemiological data. GP performed the next-generation sequencing of SARS-CoV-2 genomes and participated in the writing. NG carried out the SARS-CoV-2 structural and phylogenetic analyses and wrote the manuscript. RA and TF analyzed the data and validated mutations. ET conceived and coordinated the study. All the authors contributed to the manuscript editing and approved its final version.

## Conflict of Interest

The authors declare that the research was conducted in the absence of any commercial or financial relationships that could be construed as a potential conflict of interest.

## Footnotes

1 https://www.ecdc.europa.eu/en/coronavirus

2 https://www.iss.it/coronavirus

3 https://www.iss.it/rapporti-covid-19//asset_publisher/btw1J82wtYzH/content/id/5329985

4 https://nextstrain.org/ncov/

5 https://cov.lanl.gov

6 https://www.gisaid.org

7 https://www.iss.it/coronavirus

## Supplementary Material

Supplementary Figure 1 Maximum Likelihood phylogenetic analysis of Nuoro SARS-CoV-2 sequences. **(A)** Phylogenetic analysis of the 13 viral genomic sequences and SARS-CoV-2 reference genome. **(B)** Phylogenetic analysis of Nuoro SARS-CoV-2 sequences and GISAID viral genomes from Italy.
